# Preliminary results of the effect of music therapy treatment on anxiety, sadness, physical discomfort, mood, and quality of life in hospitalized onco-haematological patients

**DOI:** 10.1186/s40359-023-01459-x

**Published:** 2023-12-05

**Authors:** Patricia Martí, Irene Fontanals, Joel Funtané, Cristina Canaletas, Jorge Sierra, Mariona Rodés, Melissa Mercadal-Brotons, Iria González, Silvana Novelli

**Affiliations:** 1 Escola Superior de Música de Catalunya - ESMUC, Barcelona, Spain; 2 Fundació Lliga Catalana d’Ajuda Oncològica - Oncolliga, Barcelona, Spain; 3 Hematology Department, Hospital de la Santa Creu i Sant Pau, Barcelona, Spain; 4 Institut de Recerca Hospital de La Santa Creu i Sant Pau, Barcelona, Spain

**Keywords:** Music therapy, Leukaemia, Lymphoma, Transplantation, Anxiety, Sadness, Physical discomfort

## Abstract

**Background:**

Physical and psychological distress may occur in patients facing an onco-haematological diagnosis and undergoing complex therapies such as intensive chemotherapy, stem cell transplantation, and immunotherapy. Studies have shown the need for incorporating different therapeutic modalities to respond to patients’ physical and psychosocial needs.

**Aims:**

The purpose of this study was to evaluate the effectiveness of music therapy treatment on mood, anxiety, depression, and physical discomfort in hospitalized onco-haematological patients.

**Methods:**

Forty patients were included in this music therapy study from November 2021 to May 2023. Treatment consisted of individual weekly music therapy sessions. Participants completed the following evaluation instruments before and after the intervention: the *Hospital Anxiety and Depression Scale (HADS)*, *Profile of Mood States—Short Form A-Version (POMS-A)*, and *European Organization for Research and Treatment of Cancer-Quality of Life Core Questionnaire-30 (EORTC QLQ-C30)*. A three-item numerical rating scale *(NRS)* for anxiety, sadness, and physical discomfort was administered at the beginning and end of each session (pre-/postsession).

**Results:**

Differences (*p* < 0.05) were shown in NRS scores for anxiety, sadness, and physical discomfort before and after the music therapy sessions. Quality of life (QoL) was affected in almost all items, and patients could be anxious at a nonclinical level, but they were clinically depressed. EORTC QLQ-C30 scores for insomnia and pain related to the hospitalization process got worse after discharge.

**Conclusions:**

The interim results of our study showed that music therapy sessions can positively change emotional distress and improve the mood of haematological patients after every session. Despite the difficulties and limitations of this study, this preliminary report contributes to a greater understanding of the potential benefits of music therapy in hospitalized onco-haematological patients.

## Background

Music has been applied in the medical-health context since ancient times, and the study and application of music as a therapeutic resource have evolved based on the beliefs and customs in each era. Interest in the application of music therapy in the medical field has been growing since the 1980s. This is a consequence of the satisfactory results of various studies and investigations. The number of scientific studies on the effects of music in medical treatment continues to grow, offering valuable insights to health care professionals and exciting implications for future research and clinical applications. This discipline has shown its beneficial effects in different fields of health, one of which is oncology [1, 2].

The application and efficacy of music therapy in the oncological context have been specifically studied and analysed during the last 30 years. Munro and Mount first described the application of music therapy in cancer patients in 1978 [3]. Since then, different studies have contributed to the knowledge and dissemination of the role of music therapy in this context, a fact that has helped to create a space and a function for music therapy in the field of oncology and psycho-oncology. Authors such as O'Callaghan and Hiscock [4] use the term "oncological music therapy" to refer to music therapy interventions aimed at cancer patients of all ages, with different cancer prognoses and at different times of the disease. Magill [5] describes integrative music therapy, a specialty of music therapy that is considered within integrative oncology programs and that is applied to treat multiple symptoms such as pain, mood disorders and aspects of communication. Music can improve pain and symptoms such as fear, anxiety, depression, frustration, and loneliness. Music therapy reduces the effects of noxious stimuli while improving the mood and producing feelings of comfort and a sense of control [6].

The literature has increasingly shown the importance of nonpharmacological and complementary therapies to control pain and other symptoms related to chronic diseases. Two meta-analyses carried out in the context of music therapy in medicine reported significant results for variables such as nausea, vomiting, mood, pain, and well-being [2, 7]. Different programs have shown how oncological music therapy is an effective complementary and therapeutic intervention offering a multimodal and comprehensive approach that makes it possible to care for a patient's physiological and psychosocial needs.

Some studies have been carried out in the context of onco-haematology. They have shown the efficacy of music therapy in improving both the physical and psychosocial well-being of bone marrow transplant patients. Among the reported benefits after participating in music therapy sessions, the reduction of anxiety, distress, and fatigue can be highlighted, as well as the increase in states of relaxation and well-being and the improvement of mood [8–17].

Music therapy interventions thus have an exciting influence on mood and can, in turn, improve quality of life. Mood and quality of life are two variables that have been the subject of interest in oncology music therapy over the years. Unfortunately, although studies exist in this field, the number of published studies with subjects with an onco-haematological diagnosis who are undergoing complex therapies (intensive chemotherapy, stem cell transplantation, immunotherapy, and palliative care) still needs to be increased.

We aimed to determine whether music therapy positively impacts mood disturbances during hospitalization in patients with haematological malignancies.

## Methods

We performed a prospective study (IIBSP-MUS-2020–137) of adult patients (≥ 18 years old) who were admitted to the haematology unit to evaluate the effect of music therapy. Patients had to be diagnosed with haematological cancer and be able to actively participate in and follow a music therapy session. Exclusion criteria were an uncontrolled psychiatric disorder and moderate or severe hearing impairment. Patients with psychological support from a psycho-oncologist were allowed to participate.

### Music therapy sessions, questionnaires, and mood assessments

#### Sessions

Weekly music therapy sessions were planned. Patients received at least one and up to six sessions during hospitalization, depending on their needs and length of hospitalization. The duration of each session ranged from 15 to 60 min and was based on the patient's musical preferences. Adherence was sometimes limited by physical discomfort and by gastrointestinal and mucosal toxicity associated with chemotherapy. Based on the results of previous studies, different therapeutic strategies were designed to improve the patient's mood and facilitate the expression of emotions and ventilation of feelings. Singing, lyric analysis, and composition were used to channel verbal and nonverbal emotions and feelings. In contrast, music-assisted relaxation was used to promote relaxation states and distract patients from their worries. The following 2 techniques were applied accompanied by both live or recorded music, according to patient preferences: Progressive Relaxation (a technique that focuses on one part of the body at a time, without creating muscular tension, and with the goal of releasing any tension on that part); and Visualization (a technique that focuses attention on expands on pleasant and calming images). Music listening and instrument playing were used to promote social interaction.

#### Questionnaires and design

Six baseline questionnaires (pretreatment) were completed as follows:

- Demographic Information Form (completed by the patient)
- Clinical Information Form (completed by the physician)
- Musical Preferences and Previous Musical Experience Questionnaire (completed by the patient)
- Hospital Anxiety and Depression Scale (HADS) (completed by the patient)
- Shortened Profile of Mood States questionnaire (POMS-A, Spanish version) (completed by the patient)
- European Organization for Research and Treatment of Cancer-Quality of Life Core Questionnaire-30 (EORTC QLQ-C30) (completed by the patient)

The following were completed before and after each music therapy session:

- Numerical Rating Scale (NRS) for Anxiety, Sadness, and Physical Discomfort (completed by the patient)
- Music Therapy Behaviour Observation Form (completed by the music therapist)

The following were completed before discharge (posttreatment 1).

- Music Therapy Program Satisfaction Questionnaire (MTPSQ) (completed by the patient)
- HADS, POMS, and EORTC QLQ-C30 (completed by the patient)

The following were completed during the outpatient visit 2 weeks after discharge (posttreatment 2).

- HADS, POMS, and EORTC QLQ-C30 (completed by the patient)

### Questionnaire parameters

The Musical Preferences and Previous Musical Experience Questionnaire collects data on patients' favourite songs, artists, musical instruments, musical styles, and the types of music and musical instruments they disliked. This questionnaire also asks about previous musical experience, band participation, or instruments played.

The HADS [18] comprises seven questions that evaluate anxiety and seven questions that evaluate depressive mood. Normal scores range between 0 and 7; risk scores range from 8–10, and scores equal to or higher than 11 are considered to indicate pathology.

The Shortened POMS-A [19] consists of a 15-item, five-point adjective rating scale in which participants rate how they are feeling at the current moment or during the past week (scores range from (0) “not at all” to (4) “extremely”). This self-report inventory includes four negative affect scales (the Tension, Depression, Anger, and Fatigue scales) and one positive affect scale (the Vigour scale). Subscale scores are summed to determine the total mood disturbance score (TMS), for which higher scores indicate greater mood disturbances.

The *European Organization for Research and Treatment of Cancer-Quality of Life Core Questionnaire-30, EORTC QLQ-C30* [20, 21] is a questionnaire used to assess the quality of life of cancer patients, and it is a typical test used in the music therapy field. It includes five multi-item scales of functioning (physical, role, social, emotional, and cognitive functioning), a Global Health Scale, and a Scale of Symptoms and Problems. Twenty-eight of the 30 items are measured by a four-point Likert-type scale (1 = not at all, 2 = a little bit, 3 = quite a bit, 4 = very much), and the two remaining items, which address global physical health and global quality of life, are measured by a seven-point scale. The time frame focuses on the previous week. The raw scores for all multi and single-item scales are linearly transformed to 0-to-100 scales, with '100' reflecting the highest functioning or highest symptomatology. Our population included different types of haematological patients, and we considered it more appropriate to compare them with an "all cancer patients” referral sample [20].

At the end of the program, patients anonymously completed the *Music Therapy Program Satisfaction Questionnaire (MTPSQ)*, designed ad hoc for this study, to provide information about their personal experiences in the music therapy program.

### Statistical analysis

We used frequencies and dispersion statistics to describe the study population and scale values. To analyse differences before and after music therapy sessions using the NRS for Anxiety, Sadness, and Physical Discomfort, we performed a two-tailed nonparametric paired sample test (Wilcoxon's signed rank test). We also used an unpaired two-sample t test to compare patients’ EORTC QLQ-C30 scores with those of the referral population. We performed a power analysis with an effect size of 1 and alpha error of 0.05 to determine which parameters could be compared with a power of detection ≥ 80%. Due to the sample size (the number of patient questionnaires), the only questionnaires that could be compared were the NRS (first, second, and third sessions), POMS-A (baseline and after discharge), and HADS (baseline and after discharge). The results of the other questionnaires (EORTC-Q30, POMS predischarge, and HADS predischarge) could not be compared due to the size of the sample and the risk of losing power to detect differences. Despite this, we will report the results.

We used GraphPad Prism Version 9.5.1; RStudio 2022.07.0. We used the statistical graphical user interface EZR [22], PROscorer, and the QoLMiss package [23, 24].

## Results

**Fig. 1:**
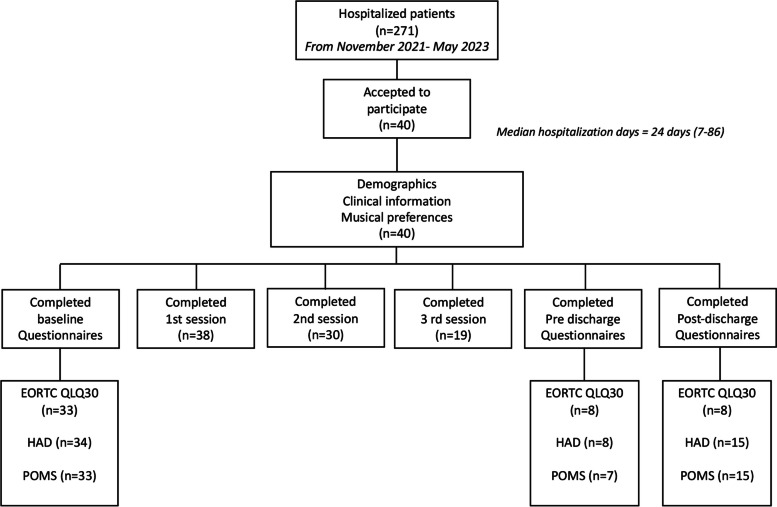
Consort flowsheet participants

We present the preliminary results of the first 40 patients included in the study from November 2021 to May 2023 (see Fig. 1. Consort flowsheet participants). All participants gave their informed consent.

The patients' characteristics are summarized in Table 1. Three patients were taking anxiolytics (alprazolam), and one of them was also taking an antidepressant (citalopram). Two patients received psychological support.

**Table 1:** Patient characteristics

Variable		Frequency	Percentage (%)
Gender	Male	18	45
	Female	21	52.5
	Nonbinary	1	2.5
Age (years)	48.22 (median)	(20–73) range	
Civil status	Single	16	40
	Married	19	47.5
	Living with a partner	3	7.5
	Divorced	1	2.5
	Widowed	1	2.5
Siblings	Yes	23	57.5
Study level	Basic degree	3	7.5
	Mid-degree	13	32.5
	Higher degree	24	60
Disease Stage	Advanced Disease	15	37.5
Chemotherapy	Acute Myeloid Leukaemia	12	30
	Acute Lymphoblastic Leukaemia	6	15
	Non-Hodgkin’s Lymphoma	6	15
	Hodgkin’s Lymphoma	3	7.5
	Autologous Stem Cell Transplant	12	30
	Allogeneic Stem Cell Transplant	1	2.5

### Adherence to the protocol

Despite offering the study to all potential participants, only 15% agreed to participate. We perceived that adult patients did not fully understand the concept of music therapy and that they preferred not to experience disturbances that could break their equilibrium. The NRS for Anxiety, Sadness, and Physical Discomfort before and after each music therapy session was completed by ≈100% of the participants. However, the HADS, POMS-A, and EORTC QLQ-C30 questionnaires were mainly completed at baseline. Less than half of the patients with more than one music therapy session completed questionnaires before discharge and in the outpatient setting.

**Fig. 2:**
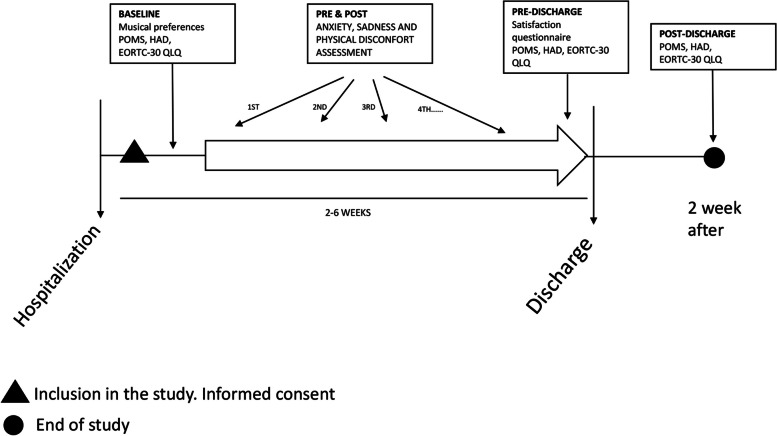
Study design

The study was conducted as described in Fig. 2.

### Numerical rating scale (NRS) for anxiety, sadness and physical discomfort

The NRS is a continuous scale with a total score up to 10 points. The distribution of the samples was not normal (Shapiro‒Wilk test). Wilcoxon's signed rank test showed differences (*p* < 0.05) in the anxiety, sadness, and physical discomfort scores before and after the music therapy sessions. The positive effect of music therapy was similar after the first, second, and third sessions. In general, the reduction in symptoms was ≥ 50%. The results are summarized in Table 2. The effect was consistent for every music therapy session. To analyse whether there was a tendency over time, we performed a Friedman test, and we did not find any tendency.

**Table 2:** Numerical rating scale for anxiety, malaise, and sadness

	pre (mean)	post (mean)	Reduction (%)	Statistic^a^	p	Mean difference	SE difference	Lower	Upper
Before and After the First Session (*n* = 38)									
Anxiety	2.711	1.237	54	413.5	< 0.001	2	0.258	1.5	2.5
Sadness	3.5	1.289	63	325	< 0.001	3	0.394	2	4
Physical discomfort	3.974	1.974	50	402	< 0.001	2.5	0.337	2	3.5
Before and After the Second Session (*n* = 30)									
Anxiety	2.833	1	65	296	< 0.001	2	0.322	1.5	2.5
Sadness	2.933	1.133	61	253	< 0.001	2	0.334	1.5	3
Physical discomfort	3.7	1.633	56	325	< 0.001	2	0.339	1.5	3
Before and After the Third Session (*n* = 19)									
Anxiety	2.211	0.684	69	105	< 0.001	2	0.362	1	3
Sadness	2.474	0.526	79	120	< 0.001	2	0.487	1.5	3
Physical discomfort	3.000	1.000	67	91	0.002	2.5	0.496	1.5	4

^a^Nonparametric Wilcoxon test

### POMS and HADS

Thirty-three patients completed the POMS-A at baseline [25]. The mean value for anger was 1.00 (SD 2.26), that for fatigue was 4.0 (SD 3.17), that for vigour was 5.0 (SD 2.78), that for tension was 3.0 (SD 2.18), and that for depressed mood was 2.0 (SD 2.69). The mean POMS-A total mood disturbance score was 21.0 (SD 9.31).

Seven patients completed the POMS-A predischarge. The mean value for anger was 2.00 (SD 3.25), that for fatigue was 6.0 (SD 3.34), that for vigour was 5.0 (SD 2.37), that for tension was 3.0 (SD 2.58), and that for depressed mood was 2.0 (SD 2.24). The mean POMS-A total mood disturbance score was 23.0 (SD 10.5).

Fifteen patients completed the POMS-A 2 weeks after discharge. The mean value for anger was 4.00 (SD 2.34), that for fatigue was 3.0 (SD 3.02), that for vigour was 5.0 (SD 2.63), that for tension was 3.0 (SD 2.64), and that for depressed mood was 3.0 (SD 2.87). The mean POMS-A total mood disturbance score was 23.0 (SD 8.9).

The maximal expected score for the POMS-A was 60 (with 0 indicating no mood disturbance, 30 indicating a moderate mood disturbance, and 60 indicating an extreme mood disturbance).

With these results, we can conclude that there is a tendency towards moderate mood disorder in these patients.

Thirty-four patients completed the HADS questionnaire at baseline [18]. This revealed a mean score of 7 (SD 2.30) for anxiety and 13.2 (SD 1.92) for depression.

Eight patients completed the HADS questionnaire predischarge. This revealed a mean score of 8 (SD 3.31) for anxiety and 14.5 (SD 3.59) for depression.

Fifteen patients completed the HADS questionnaire two weeks after discharge. This revealed a mean score of 8 (SD 2.95) for anxiety and 13.0 (SD 2.85) for depression.

In summary, patients provided risk scores for anxiety predischarge and two weeks after discharge, and there were pathological scores across all times for depression.

**Fig. 3:**
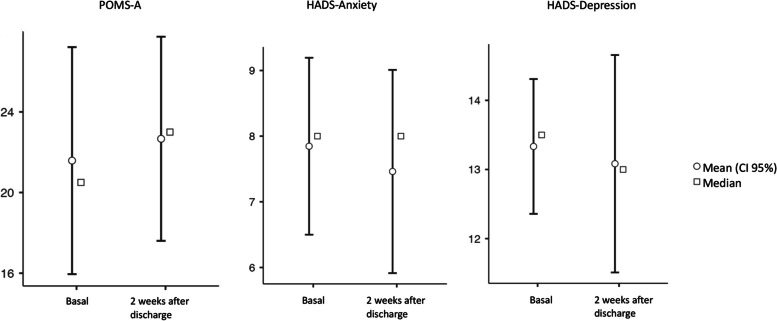
POMS-A and HAD score

To determine the presence of differences between POMS-A, HADS anxiety, and HADS depression scores at baseline and two weeks after discharge, we performed Wilcoxon's signed rank test, and we did not find any differences (see Fig. 3). We did not include predischarge questionnaires because the number of completed questionnaires was not enough to make comparisons.

### EORTC QLQ-C30 questionnaire

The results of the EORTC QLQ-C30 in our study population are summarized in Table 3.

**Table 3:** EORTC QLQ-C30

**BASELINE (*n* = 33)**	**Mean**	**Median**	**SD**	**Minimum**	**Maximum**
Global Health Status score	54.8	58.3	22.9	8.33	100.0
Role Functioning Score	43.2	33.3	37.8	0.00	100.0
Physical Functioning Score	60.8	66.7	28.1	6.67	100.0
Emotional Functioning score	67.4	75.0	22.0	16.67	91.7
Cognitive Functioning Score	65.7	66.7	28.8	0.00	100.0
Social Functioning Score	35.9	33.3	30.9	0.00	100.0
Fatigue Score	46.9	44.4	29.8	0.00	100.0
Nausea & Vomiting Score	79.2	83.3	24.7	16.67	100.0
Pain Score	57.8	66.7	29.6	0.00	100.0
Dyspnoea Score	91.7	100.0	18.9	33.33	100.0
Insomnia Score	57.6	66.7	31.5	0.00	100.0
Appetite Loss Score	53.5	66.7	37.2	0.00	100.0
Constipation Score	72.7	100.0	34.8	0.00	100.0
Diarrhoea Score	72.7	100.0	34.8	0.00	100.0
Financial Difficulties Score	80.8	100.0	30.1	0.00	100.0
**PREDISCHARGE (*n* = 8)**	**Mean**	**Median**	**SD**	**Minimum**	**Maximum**
Global health status score	50.0	50.0	11.8	33.3	66.7
Physical functioning score	56.2	46.7	27.4	20.0	100.0
Role functioning score	33.3	33.3	31.6	0.0	66.7
Emotional functioning score	66.7	66.7	16.1	33.3	83.3
Cognitive functioning score	66.7	75.0	29.5	33.3	100.0
Social functioning score	56.3	66.7	21.7	16.7	83.3
Fatigue score	52.4	55.6	27.0	22.2	100.0
Nausea & vomiting score	73.8	83.3	30.2	33.3	100.0
Pain score	54.8	50.0	38.1	0.0	100.0
Dyspnoea score	90.5	100.0	16.3	66.7	100.0
Insomnia score	47.6	66.7	37.8	0.0	100.0
Appetite loss scale	47.6	33.3	42.4	0.0	100.0
Constipation score	61.9	66.7	40.5	0.0	100.0
Diarrhoea score	57.1	33.3	41.8	0.0	100.0
Financial difficulties score	66.7	66.7	35.6	0.0	100.0
**POSTDISCHARGE (*n* = 13)**	**Mean**	**Median**	**SD**	**Minimum**	**Maximum**
Global Health Status Score	47.4	50.0	22.1	16.7	83.3
Role Functioning Score	48.7	50.0	36.9	0.0	100.0
Physical Functioning Score	70.3	80.0	26.6	20.0	100.0
Emotional Functioning Score	63.5	66.7	25.8	16.7	100.0
Cognitive Functioning Score	73.1	83.3	25.9	16.7	100.0
Social Functioning Score	46.2	50.0	28.2	0.0	100.0
Nausea & Vomiting Score	84.6	83.3	18.6	50.0	100.0
Fatigue Score	54.7	55.6	29.2	11.1	100.0
Pain Score	78.2	83.3	24.9	16.7	100.0
Dyspnoea Score	100.0	100.0	0.0	100.0	100.0
Insomnia Score	74.4	66.7	27.7	0.0	100.0
Appetite Loss Score	64.1	66.7	37.2	0.0	100.0
Constipation Score	94.9	100.0	12.5	66.7	100.0
Diarrhoea Score	82.1	100.0	32.2	0.0	100.0
Financial Difficulties Score	82.1	100.0	25.9	33.3	100.0

We performed a Wilcoxon's signed rank test between baseline and postdischarge scores, and we found a significant increase in pain and insomnia scores after discharge (*p* < 0.05).

We also compared the baseline EORTC QLQ-C30 (*n* = 33) with the "all cancer patients" referral population (see Table 4).

**Table 4:** Comparisons against the referral population

	Baseline		All cancer patients		
	mean	SD	**mean**	**SD**	p^a^
Global Health Status Score	54.8	22.9	61.3	16.8	*0.0264*
Role Functioning Score	43.2	37.8	76.7	21.5	*0.001*
Physical Functioning Score	60.8	28.1	70.5	22.4	*0.031*
Emotional Functioning Score	67.4	22.00	71.4	19.4	0.2367
Cognitive Functioning Score	65.7	28.8	82.6	22.5	*0.001*
Social Functioning Score	35.9	30.9	75.00	21.4	*0.001*
Fatigue Score	46.9	29.8	34.6	22.7	*0.0019*
Nausea & Vomiting Score	79.2	24.7	9.1	14.5	*0.001*
Pain Score	57.8	29.6	27.00	24,00	*0.001*
Dyspnoea Score	91.7	18.9	21.00	20.7	*0.001*
Insomnia Score	57.6	31.5	28.9	28.00	*0.001*
Appetite Loss Score	53.5	37.2	21.1	19.9	*0.001*
Constipation Score	72.7	34.8	17.5	24.1	*0.001*
Diarrhoea Score	72.7	34.8	9.00	18.1	*0.0001*
Financial Difficulties Score	80.8	30.1	16.3	20.7	*0.0001*

^a^Unpaired T test, two-tailed

For the functioning scale and global QOL scores, our population had impaired functioning compared with the referral population, except for emotional functioning. For the symptom scales, our patients had higher scores, indicating a higher symptom burden.

### Music therapy program satisfaction questionnaire (MTPSQ)

Fifteen patients completed the MTPSQ before discharge.

**Fig. 4:**
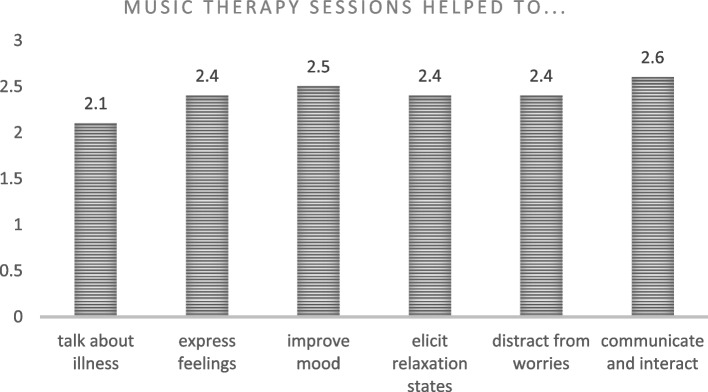
Patients’ general mood perceptions. [0= Not at all; 1= A little; 2= Enough; 3= A lot]

With regard to the patients’ general mood perceptions within the music therapy sessions, the results showed a mean score of 2.5 (based on a Likert scale ranging from 0 to 3) (see Fig. 4).

This questionnaire permitted us to explore other aspects that may have contributed to elevating the patients’ moods, since they deal with different strategies and resources that may help in emotional self-regulation. Communicating and interacting with music therapists, family caregivers, and staff was considered the best strategy during the music therapy sessions, with a mean score of 2.6. The expression of emotions and feelings, distractions and relaxation activities were perceived as a favourable strategy, with a mean score of 2.4. Speaking about one’s illness was an important issue addressed during the sessions that might have also led to facilitating emotional expression and ventilation, with a mean score of 2.1.

Based on patient ratings, we can affirm that the participants perceived this program as useful, with a mean score of 2.66 (0 = not at all; 1 = a little; 2 = enough; 3 = a lot).

Finally, it is important to state that almost all of the participants would recommend this program to other patients; a mean score of 2.93 was obtained for this item.

## Discussion

Our study was designed to answer two main research questions: (a) what are the possible changes in the moods, anxiety, depression, and quality of life of hospitalized haematological cancer patients after music therapy (pre-/posttreatment), and (b) what are the possible changes during each 15/60-min music therapy session (pre-/postsession) in the perceived anxiety, sadness and physical discomfort of haematological cancer patients?

The main difference between our study and previous reports is that it includes hospitalized patients only, a complex population (the majority isolated), and our intervention is adapted to each patient with a particular clinical condition.

Here, we present the first preliminary results of the study, which reflect a complex population with diminished QoL and clinically relevant mood disorders.

Less than 20% of the patients agreed to participate. This avoidance might reflect the difficulties and insecurities that these patients were experiencing during the particular clinical episode.

It is even possible that the patients who agreed to participate were those with a better status; this is worrying since the patients who did not participate were not receiving any psychological help. Therefore, it is imperative to actively implement psychological support for this population.

This study compared the effect of music therapy on patients’ mood from pre- to post-session based on three parameters (anxiety, sadness, and physical discomfort) that changed significantly. We demonstrated that music therapy was effective in reducing patients’ emotional and physical discomfort during the sessions, helping to alleviate anxiety, sadness, and physical discomfort every time it was delivered.

As clinicians are directly involved in therapeutically treating and supporting these patients, we consider it relevant to determine the number or type of interventions that could be combined with music therapy to achieve better control of symptoms in the future. This is a matter of frequency and would depend on the ability to adapt music therapy sessions to the clinical scenario. Treatment adherence to music therapy and continuous attendance to following sessions of this group matches with attendance rates of other studies with patients who are in active oncologic treatment, where participants have lower attendance rates or even abandon programs due to health complications, deterioration of symptoms, and physical limitations, as well as referral to intensive care unit or even death. Many music therapy studies in oncology have based the intervention on merely one or two sessions, and few offer a continuity of more sessions.

Achieving the ideal music therapy dosage might also impact the global perception of all supportive therapies that could improve mental health. It is essential to show that these interventions are relevant to patients experiencing the oncological process with tangible results. The knowledge that music therapy is effective will help improve adherence and inspire others to participate in music therapy programs. The participants' satisfaction was high, and no participants reported adverse effects.

We are very aware that the improvement of QoL is linked to the restoration of health; in our case, this would be the resolution of the oncological process. That is why we were not surprised that we could not improve QoL with music therapy during hospitalization, even less so in a short time.

However, if music therapy (and other supportive interventions) is maintained over time, particularly in outpatient settings, QoL could be improved. The objective is to accompany the patient and make the process more bearable, even in an end-of-life situation.

This study has several limitations. We are reporting our first data, which represents an intent-to-treat population. This study included a heterogeneous population, but it perfectly reflected the reality of more severely compromised haematological patients, and with this first analysis, we were able to establish the future direction we must take.

## Conclusions

This study indicated that music therapy can positively change emotional distress and improve the moods of haematological patients. Despite the difficulties and limitations of this study, the authors hope this paper contributes to a greater understanding of the potential benefits of music therapy in this population.

## Data Availability

Data are available on request only due to ethical & legal reasons.

## Abbreviations

EORTC QLQ-C30: European Organization for Research and Treatment of Cancer-Quality of Life Core Questionnaire-30
HADS: Hospital Anxiety and Depression Scale
MTPSQ: Music Therapy Program Satisfaction Questionnaire
NRS: Numerical Rating Scale
POMS-A: Shortened Profile of Mood States questionnaire
QoL: Quality of life
SD: Standard deviation
TMD: Total mood disturbance score
